# Pseudomonas-Enterobacter Co-Infection Drives Cellulitis and Lymphangitis in Equines: A Case Report

**DOI:** 10.3390/vetsci12060574

**Published:** 2025-06-11

**Authors:** Xiangning Huang, Renjie Deng, Haoen Huang, Huisheng Xie, Aolei Chen

**Affiliations:** 1 College of Veterinary Medicine, South China Agricultural University, 483 Wushanlu, Guangzhou 510640, China; nelliehuangxn@stu.scau.edu.cn (X.H.);; 2 Guangdong Technological Engineering Research Center for Pet, 483 Wushanlu, Guangzhou 510640, China; 3 Chi University, 9650 West Highway 318, Reddick, FL 32686, USA; shen@chiu.edu

**Keywords:** horse, lymphocutaneous infections, *Pseudomonas asiatica*, *Enterobacter hormaechei*, traditional Chinese veterinary medicine (TCVM)

## Abstract

This case report detailed a rare co-infection of *Pseudomonas asiatica* and *Enterobacter hormaechei* in a 9-year-old warmblood mare, leading to severe cellulitis and secondary lymphangitis following traditional hoof blood-letting therapy. The mare exhibited acute limb swelling, fever, cutaneous ulceration, lymphatic dysfunction and unknown anemia. Comprehensive diagnostics, including bacterial culture, whole-genome sequencing, anti-elastin antibody (AEAb) ELISA, and diagnostic imaging, confirmed the pathogens causing cellulitis and secondary lymphangitis. AEAb levels were elevated, correlating with lymphatic degradation, while radiography and lymphangiography ruled out laminitis but identified tortuous lymphatic vessels. The treatment integrated systemic antimicrobials, anti-inflammatory therapy, combined decongestive therapy, and traditional Chinese herbal medicine, resulting in resolution of infection, improved hematological parameters, and restored athletic performance. The therapeutic regimen primarily included gentamicin, enrofloxacin, oxytetracycline, and the Wei Qi Booster. The case highlights the critical role of pathogen-directed antimicrobial selection and the potential benefits of combining conventional and holistic therapies. This report emphasizes the necessity of early, multifaceted interventions to prevent life-threatening complications in equine cellulitis–lymphangitis cases.

## Simple Summary

Cellulitis/lymphangitis is a common disease in horses. Clinicians diagnose these diseases based on empirical judgement, namely the clinical presentation such as fever, limb swelling and acute lameness. This may lead to misdiagnoses or missed diagnoses or even practice against antibiotic stewardship. However, there are limited published reports focusing on diagnostic tests and integrative treatment regarding cellulitis/lymphangitis. This case report first documented a comprehensive approach using bacterial culture, bacterial genomics analysis and lymphangiograms to confirm the diagnosis.

## 1. Introduction

Cellulitis is an acute infection of the skin and subcutaneous tissues that is typically caused by bacteria. In most cases we cannot identify the bacterial pathogens, with many negative cultural results turning out to be due to the previous use of antibiotics [1]. Epidemiologically, cellulitis represents a common condition, affecting individuals across various age groups, and its incidence is particularly high among leisure horses and sports horses [2]. Although most cases respond well to appropriate antimicrobial therapy, complications—including sepsis, abscess and secondary lymphangitis—can adversely affect the prognosis. Poor prognosis may lead to motor dysfunction and, worse still, euthanasia [3,4,5]. On the other hand, lymphangitis is inflammation and/or infection of the lymphatic vessels [6]. Clinically, lymphangitis shares a similar presentation to cellulitis, which sometimes makes these two words interchangeable.

Common clinical signs of cellulitis include localized erythema, swelling, pain, and fever [3,4]. However, these features broaden the clinical spectrum of the disease and emphasize the need for careful and comprehensive evaluation. Currently, the clinical diagnosis of cellulitis basically relies on the evaluation of characteristic symptoms and the clinician’s empirical judgment [4,7]. Therefore, reliance on subjective assessment can lead to misdiagnoses or missed diagnoses.

The standard treatment regimen for cellulitis typically includes a combination of systemic antibiotic therapy, supporting treatment, and in severe cases, incision and drainage [8,9]. According to the study of Adam and Southwood, pain management is also an important factor that affects the therapeutic outcome [10]. However, various severe complications such as avulsion of the hoof capsule, dermal necrosis, laminitis, and widespread thrombosis and permanent lameness may lead to a decrease in the survival rate [10,11,12]. Therefore, timely diagnosis, comprehensive evaluation, early intervention and tailor-made treatment plans are essential to improve prognosis.

This case describes the clinical manifestation, diagnosis and treatment of lymphangitis secondary to cellulitis in a 9-year-old warmblood mare caused by *Pseudomonas asiatica* and *Enterobacter hormaechei* coinfection. This case report highlights the multimodal diagnostic methods and the support given to the animal in a holistic manner.

## 2. Materials and Methods

### 2.1. Case Description

A 9-year-old warmblood mare from an equestrian club, used for show jumping, developed swelling in the right hind limb and a high fever after traditional hoof blood-letting therapy treatment a day before presentation. This is an ancient method where an iron nail was inserted into a specific acupoint—Hou-ti-tou—to relieve inflammation and pressure. We believed improper handling was a source of infection.

**Figure 1:**
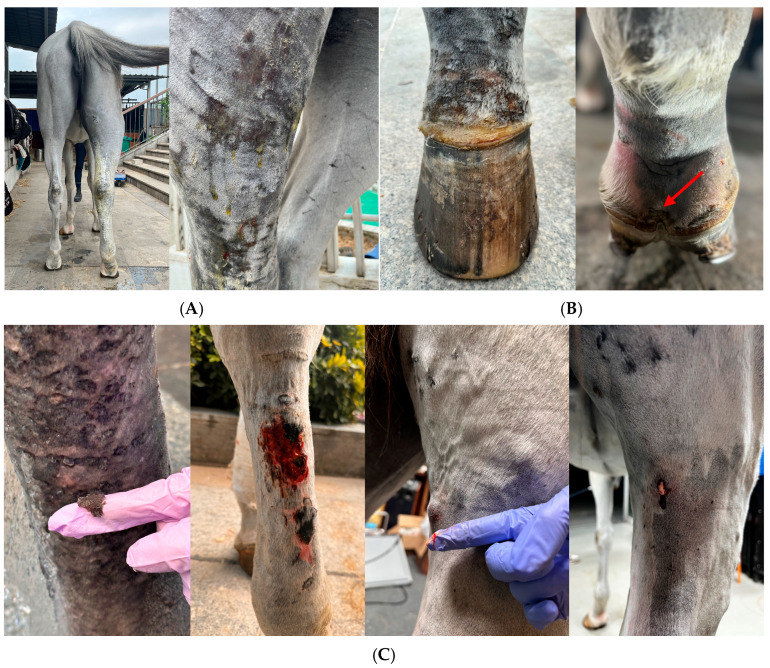
Clinical manifestation. (**A**) Acute swelling of the distal limb of the right hind leg, extending from the pastern to above the stifle joint with oozing straw-colored exudate. (**B**) Swollen coronary band of the right hind limb with mildly detached hoof wall at the plantar aspect (red arrow). (**C**) Skin sloughing, skin necrosis and ruptured pustules.

An initial physical examination revealed a high fever at 40.4 °C and the swelling gradually extended from the pastern to above the stifle joint. Pitted edema in the affected limb was noted. The skin of the affected limb became thin, erythematous, and ulcerated, and began oozing yellow exudate (Figure 1A). The coronary band was swollen, with excessive skin thickening and mild hoof-wall detachment (Figure 1B). In later stages, small pustules appeared on the skin of the gaskin and ruptured (Figure 1C).

An integrative medical treatment plan was implemented, including antimicrobial therapy and regional limb perfusion, anti-inflammation therapy, manual massage, combined decongestive therapy (CDT), and exercise rehabilitation to prevent secondary laminitis. Qi-Blood are the fundamental substances that maintain life activities and body functions in TCVM theory. The Chinese herbal medicine Wei Qi Booster was administered to tonify the Qi-Blood and strengthen immune functions, as well as the Heat for enhancing the antibiotic effects and accelerating the healing process. Gentamicin (6.6 mg/kg, I.V. q24h) and enrofloxacin (5 mg/kg, I.V. q24h) were given for 2 weeks with compression bandages and later changed to oxytetracycline (5 mg/kg, I.V. q12h) for 2 weeks when exudate recurred. Phenylbutazone (4.4 mg/kg, I.V. q12h on day 1 and decreased to 2.2 mg/kg, I.V. q12h) was prescribed to decrease inflammation and relieve pain. Manual massage was intended to help lymphatic return. In addition, the Chinese herbal medicine Wei Qi Booster (45 g, PO q12h for 10 d and 15 g, PO, q12h for 20 d), was orally given. This formula primarily contains codonopsis root (Dang Shen), astragalus root (Huang Qi), dong quai root (Dang Gui), and so on. In our 3-month follow-up, the mare was reported to be in good condition with healed skin and recovered athletic performance. In addition, the body condition score (BCS) of the mare had gone from 3/9 to 5/9.

### 2.2. Blood Examination

Blood samples were collected from the jugular vein for complete blood count (CBC) (BC-500, Mindray, Shenzhen, China), chemistry panel (Nx700i, Fujifilm, Iwate, Japan), serum amyloid A (SAA) (Vcheck-V200, Bionote, Gyeonggi-do, Republic of Korea) evaluation at South China Agricultural University Veterinary Teaching Hospital (Guangzhou, China).

### 2.3. Bacteriologic Examination

Two per cent chlorhexidine was used to clean and sterilize the skin to avoid false positives from the normal skin flora. A linear array probe was gently applied on the lateral tarsal joint region for scanning until we found the anechoic region. A 0.7 × 24 mm butterfly needle was inserted into the fluid pocket and confirmed with the ultrasound. Straw-colored exudate was collected for bacterial culture. Samples were sent to Zoetis Reference Laboratory (Shanghai, China) for bacterial culture and antimicrobial susceptibility tests using the VITEK 2 COMPACT system and the Kirby–Bauer disc diffusion method [13].

### 2.4. Bacterial Genomics Analysis

Sample 1 and Sample 2 were bacterial DNA isolated from bacterial culture. Bacterial DNA was extracted using MagPure Bacterial DNA Kit (D6361-02, Magen, Guangzhou, China). DNA concentration was determined via Qubit4.0 (Thermo, Q33226, Budapest, Hungary). DNA integrity was assessed by 1% agarose gel electrophoresis. The whole genome DNA was randomly fragmented to an average size of 200–400 bp. The selected fragments were put through end-repair, 3′ adenylated, adapter ligation, and PCR amplifying. After purification with the magnetic beads, the library was qualified by the Qubit 4.0 fluorometer and the length of library was assessed by 2% agarose gel electrophoresis. The qualified libraries were sequenced on the Illumina NovaSeq 6000 platform at Sangon Biotech (Shanghai, China). After sequencing, raw reads were filtered via Trimmomatic (v0.36) by removing adaptors and low-quality reads, then clean reads were obtained. Genome assembly was carried out using SPAdes (v3.15) and the Gapfiller (v1.11) was used for filling gaps. Gene predictions were generated using the National Center for Biotechnology Information (NCBI)nr database.

### 2.5. Anti-Elastin Antibody (AEAb) ELISA

Blood samples were obtained through the jugular vein from the animals and naturally coagulated at room temperature for 20 min. Samples were centrifugedat 2–8 °C for 20 min (2000 rpm) and the sera were carefully collected. The samples were stored at −80 °C for ELISA assay. Sera AEAb were tested by Horse ELN Ab ELISA kit (MeiMian Industrial, Jiangsu, China). The detective range for AEAbs was 10–160 ng/L. The procedure involved preparing a standard curve through sequential dilutions of the original standard (160 mg/L) to generate concentrations ranging from 10 to 160 mg/L. Samples were diluted 5-fold and added to microplate wells alongside blank controls. After incubation at 37 °C for 30 min, the plate was washed repeatedly to remove unbounded components. Horseradish peroxidase (HRP)-conjugate reagent was introduced to all wells except the blanks, followed by another incubation and washing cycle. Color development was initiated by adding chromogen substrates under light-protected conditions at 37 °C for 10 min. The reaction was terminated using a stop solution, and absorbance was measured at 450 nm within 15 min, with blank wells serving as the reference. All serum samples were repeated three times as technical replicates.

### 2.6. Survey Radiograph, Lymphangiogram and Ultrasonography

**Figure 2:**
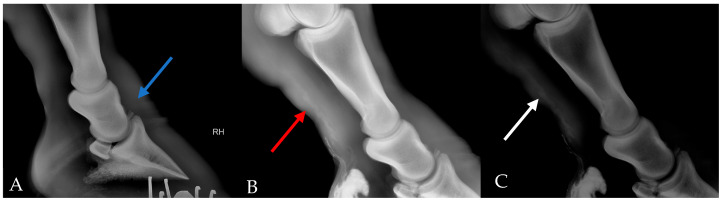
Survey radiograph and lymphangiogram of the right hind limb. (**A**) Survey radiograph of right foot, showing a bony fragment of proximal P3 (blue arrow). (**B**) Lymphangiogram showed multiple tortuous tubular structures with one specifically dilated (red arrow). (**C**) Increased contrast of B to highlight the dilated structure (white arrow).

The animal was sedated with xylazine (0.3 mg/kg). The skin was scrubbed with 2% chlorhexidine. **Survey radiograph**: Lateral and craniocaudal projections were taken in a setting of 90 kVp, 1 mA/s. **Lymphangiogram**: 10 mL of iodinated contrast medium (Iohexol, 17.5 g(I):50 mL, Cisen-Pharma, Jining, China) was injected subcutaneously at the level of the plantar aspect of the distal pastern followed by 2 mL of lidocaine (20 mg/mL) for local anesthesia. Lateral radiographs (90 kVp, 1 mA/s) of the distal extremities were recorded 10 min after the injection and manual massage (Figure 2). However, due to difficult manipulation of the animal, only 2 mL of contrast medium was successfully injected. **Ultrasonography:** a brief ultrasound examination of the distal limb using a Vetus E7pro ultrasound machine (Mindray Animal Medical Technology, Shenzhen, China) with a 7–13 MHz linear array transducer.

### 2.7. Data Analysis

AEAb levels were expressed as optical densities (OD’s). Relative expression levels are presented as mean ± standard deviation (SD). The statistical significance of differences was evaluated with the Welch’s t-test using GraphPad Prism v10.0.1 software (* *p* <0.05).

## 3. Results

### 3.1. Blood Work Results

Laboratory results showed inflammatory leukogram and mild regenerative anemia initially. During the treatment period, leukocytosis was greatly controlled while the anemia still existed for unknown reasons. Serum amyloid A (SAA) declined to normal indicating the acute inflammation period had passed. At the same time, serum biochemistry showed normal liver and renal function while there was a decrease in the A/G ratio (0.5; ref. 1.5–25). The decrease in serum albumin may have contributed to the excessive lymphatic effusion. Ten days after the use of Wei Qi Booster, the anemia was resolved and the HCT level maintained at 36.3% 2 months after finishing the medication (Table 1).

### 3.2. Diagnostic Imaging Results

An X-ray was performed to rule out other musculoskeletal diseases that could have caused acute swelling in the right hind limb. The lateral view of the survey radiograph showed soft tissue swelling and normal bone structures except for a bony fragment of proximal distal phalanx (P3), which previously existed and caused no lameness. No signs indicated potential secondary laminitis.

A lymphangiogram revealed several dilated tortuous lymphatic vessels in the plantar aspect of the distal limb (Figure 2). Ultrasound examination revealed marked subcutaneous and fascial oedema. We also noticed a hyperechoic subcutaneous tissue layer and cobblestone pattern at the coronary band indicating cellulitis.

### 3.3. Bacteriologic Examination and Pathogen Identification

Two bacterial genera, *Pseudomonas* and *Enterobacter* (phylogenetic analysis is attached in the (Figures S1 and S2), were identified as Gram-negative bacteria which are opportunistic pathogens. Both showed resistance to penicillin, cephalosporin, sulfamethoxazole, and chloramphenicol but were sensitive to gentamicin. Surprisingly, both bacteria were intermediate to enrofloxacin (Table S1). Results from bacterial genomics analysis further confirmed the pathogen species as *Pseudomonas asiatica* and *Enterobacter hormaechei*. Genomics data also revealed several multidrug-resistant genes such as *mdtE*, *mdtA*, *mdtK*, *mdtB*, *stp*, etc. This may explain why these pathogens were resistant to multiple antimicrobial categories.

### 3.4. Anti-Elastin Antibody (AEAb) ELISA Results

A total of twelve horses were sampled and analyzed, and the data were divided into the following cohorts: Group 1: the AEAb baseline level of the animal, sampled 39 days after onset; Group 2: AEAb level of the animal, re-sampled at 59 days; Group 3: AEAb level of healthy warmblood controls; Group 4: AEAb level of asymptomatic warmblood horses with chronic lymphedema; Group 5: AEAb level of healthy draft horse controls.

There was a statistically significant difference (*p* < 0.05) between Group 1 and Group 2, indicating the AEAb level may have increased as the disease progressed (Table 2); Group 5 showed statistically significant differences compared to Group 3 (*p* < 0.05), indicating the AEAb level may have breed difference (Table 3). Group 4 had statistically significant differences compared to Group 3 (*p* < 0.05) combined with the fact that the mean AEAb level of the animals (Group 1 and Group 2) is higher than warmblood horses with chronic lymphedema (Group 4), indicating that the AEAb level increased in affected horses.

## 4. Discussion

Cellulitis is a common disease in horses. It can be caused by a tiny penetration and, without proper management, can lead to severe complications like endocarditis, osteomyelitis, and toxic shock [14]. Therefore, timely diagnostic examinations and early therapeutic interventions are crucial to prevent serious complications. Cellulitis can present with a wide range of symptoms, including swelling, localized fever, pain, and oozing serum. Some clinical signs are common and may be confused with other diseases, while some clinical signs are rare. A previous case report described hindlimb cellulitis that triggered lymphangitis, with bacterial cultures yielding *Staphylococcus aureus* and *Escherichia coli*. In the later stages of the disease, detachment of the hoof capsule occurred, ultimately resulting in euthanasia of the horse [11]. This case report documented some uncommon symptoms of cellulitis such as an extensive lymphedema region along with pustules, skin sloughing, and skin ulceration, and unknown anemia. From a TCVM perspective, Qi serves as the body’s primary defensive mechanism, preventing the invasion of pathogens, including bacteria. When Qi is deficient, this protective barrier is weakened, allowing bacteria to enter the body more easily and cause infections. TCVM also believes that the Blood is generated from Qi. Therefore, insufficient Qi can impair the Blood production, ultimately leading to anemia.

*P. asiatica* and *E. hormaechei* are both opportunistic pathogens that can inhabit wet environments including water and soil [15,16]. *P. asiatica* is a newly identified species firstly isolated in Japan that seldom causes severe infections unless the host went through invasive procedures or was immunocompromised [17]. No disease caused by *P. asiatica* in horses has been reported to date, which may be partially attributed to the lack of bacterial identification in clinical practice. *E. hormaechei* is a member of the Enterobacter cloacae complex (ECC) which often leads to severe nosocomial infections through its multidrug resistance. Research showed that *E. hormaechei* is more toxic compared to other ECC members, and can cause cellulitis as well as pneumonia, urinary infections, and so on [18]. They both share an efflux pump genes mechanism, along with the activity of β-lactamase production, jointly contributingto their multidrug resistance. It is worth noting that both strains have been implicated in nosocomial outbreaks in human hospitals [15,19,20]. In 2022, researchers in China isolated *Pseudomonas putida* strains, a strain close to *P. asiatica*, harboring five distinct β-lactamases, highlighting the escalating threat of antibiotic-resistant bacteria as a critical public health issue spanning both veterinary and human medicine [21].

Chronic progressive lymphedema (CPL) is thought to be a genetic disease that commonly affects draft horses and shares a similar clinical presentation with cellulitis. This disease is characterized by progressive swelling of the distal limbs, accompanied by scaling, marked dermal fibrosis, and the development of skinfolds and nodules, which are often complicated by secondary infections [22]. Under pathological circumstances elastin is increasingly degraded into peptides (elastin derived peptides: EPs), which are released into the circulation. The immune system initiates an immune response against the released peptides, generating anti-elastin antibodies (AEAb) [23]. Elastin is a major supportive component of lymphatics, and thus essential for their function, while elevated levels of AEAb suggest the occurrence of lymphatic pathology [24]. This study revealed significantly elevated AEAb levels in a warmblood horse affected by lymphangitis. Although the clinical signs improved, the animal’s AEAb level kept increasing. This may be due to a 70-year half-life of AEAbs [25]. As a result, we conclude that there is a breed-specific difference in AEAb levels, which is inconsistent with what Van Brantegem proposed [26]. Risk factors such as age, gender, pregnancy status, and laboratory differences can affect AEAb levels [27]. Previous studies have explored whether the ELISA test can be used as an indicator to diagnose CPL. A study revealed that the ELISA technique used to identify serum levels of AEAb was a valuable tool for the diagnosis of CPL and associated with the severity of the disease, while another study showed that ELISA procedure was not useful for CPL detection due to low sensitivity [26,27]. Brys M proposed a multimodal-integrated diagnostic approach to confirm CPL [22]. Although our animal exhibited elevated AEAb levels, she did not completely fit into the diagnostic criteria for CPL. According to our data, we believe that AEAb levels can be used as a conjunctive test for lymphangiopathy. However, the disadvantage of this study was the lack of a sufficient number of samples. Further research is needed on whether AEAbs can be used as an indicator of lymphangitis.

**Figure 3:**
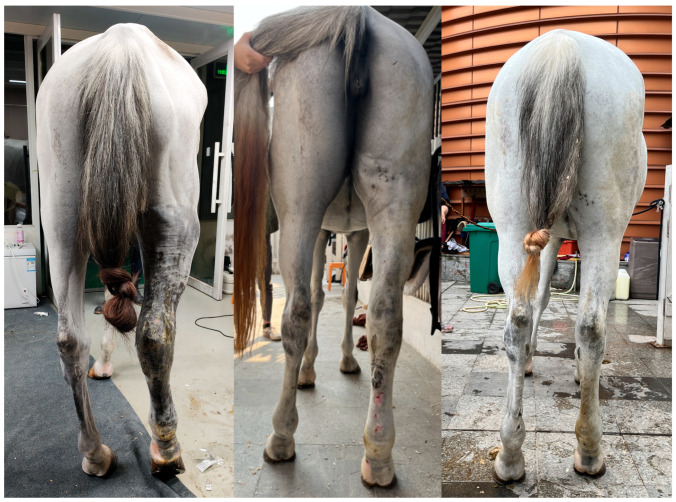
Clinical improvement. From left to right: initial presentation, 6-week follow-up, 5-month follow-up.

In this case, a holistic treatment approach was adopted, including antimicrobial and anti-inflammatory therapy, and traditional Chinese medicine, pain control, and CDT. CDT is a treatment for human lymphoedema. It can be an effective treatment for limbs swelling in horses which consists of phase I and phase II. Phase I involves daily manual lymph drainage (MLD), skin care, multi-layer short-stretch bandaging, and exercise to rapidly reduce edema and soften fibrotic tissue until the limb volume stabilizes. Phase II focuses on maintenance through continued skin care, exercise, and a transition to elastic compression garments, with MLD tapered to minimal frequency, ensuring long-term prevention of fluid re-accumulation [28]. Pain control was of paramount importance during treatment, and exercise played a critical role in recovery. Routine care included mild washing after exercise—strictly avoiding irritation and refraining from vigorous scrubbing. Considering the risk of laminitis and hoof capsule detachment, an X-ray was implemented and the result showed no evidence of laminitis. With strategic management, we resolved the potential severe complications and hoof capsule detachment, and the animal showed a good condition by a 5-month follow-up (Figure 3).

In the later phase of treatment, the traditional Chinese medicine Wei Qi Booster was used to address persistent unknown anemia and lymphedema. In TCVM, Qi is responsible for the movement of body fluids. When Qi is deficient, this physiological function is impaired, leading to the accumulation of fluids, which may manifest as edema, including lymphedema. Qi also plays a vital role in the generation of the Blood, which nourishes all tissues, including the skin. Therefore, tonifying Qi can support skin healing and overall tissue regeneration. Exploring the appropriate use of traditional Chinese medicine may be an adjunct therapy for cellulitis and lymphangitis in the future.

Although our multidimensional data provides a diagnostic reference framework for similar cases, several limitations persist. First, the application of diagnostic imaging examinations, especially the ultrasound scan, is highly dependent on the skill level of the operator. We should also consider the availability of advanced equipment in clinical settings. Additionally, the prohibitively increased cost of comprehensive diagnosis must be acknowledged. To address these challenges, we recommend prioritizing bacterial isolation and identification alongside antimicrobial susceptibility testing to facilitate timely initiation of antibiotic therapy. Subsequent diagnostic steps, such as diagnostic imaging or an AEAb ELISA test, should be tailored to the patient’s clinical progression and the owner’s financial capacity, ensuring a balance between diagnostic rigor and practical feasibility. While the herbal formula used in this case demonstrated efficacy in the affected animal, its mechanisms of action still lack support in the current literature. When applied to different clinical presentations, a thorough TCVM diagnostic evaluation should precede the selection of herbal therapies to ensure treatment alignment with specific pathological manifestations.

## 5. Conclusions

This case report presents a 9-year-old warmblood mare diagnosed with lymphangitis secondary to cellulitis caused by a rare *Pseudomonas asiatica* and *Enterobacter hormaechei* coinfection. This report highlights that comprehensive diagnostic workups must precede empirical treatment to prevent life-threatening complications like hoof capsule detachment. Notably, combining antibiotic treatment with traditional Chinese herbal therapy, pain management, and CDT can achieve a better prognosis.

## Figures and Tables

**Table 1:** Blood work results.

	10.25	10.27	10.30	11.02	11.16	12.23	3.15	Units	Reference Range
RBC	5.68	5.49	6.65	5.9	5.59	9.65	7.92	10^12^/L	5.3–10.5
HGB	88 ↓	84 ↓	101	89 ↓	86 ↓	155	131	g/L	100–170
HCT	24.2 ↓	21.7 ↓	26.7 ↓	23.6 ↓	22.9 ↓	40.1	36.3	%	30–49
WBC	22.24 ↑	19.11 ↑	11.24	13.18 ↑	7.47	10.70	7.79	10^9^/L	5.00–12.00
Lymphocytes	1.27 ↓	1.8	2.39	2.16	2.70	3.25	3.00	10^9^/L	1.32–5.86
Neutrophils	20.15 ↑	15.78	8.40 ↑	10.44 ↑	4.02	6.73	4.24	10^9^/L	2.18–6.96
Monocytes	0.81	1.32 ↑	0.35	0.50	0.38	0.37	0.31	10^9^/L	0.05–0.92
NEUT%	90.6 ↑	82.6 ↑	74.8 ↑	79.3 ↑	53.8	62.9	54.4	%	38.0–70.0
LYMPH%	5.8 ↓	9.4 ↓	21.3 ↓	16.4 ↓	36.1	30.4	38.5	%	25.0–62.0
Serum amyloid A(SAA)	/	/	101.2	/	<5	/	/	Ug/mL	<10
Albumin	/	/	/	24.0 ↓	23.0 ↓	30 ↓	/	g/L	40–55
Globulin	/	/	/	53.0 ↑	46.0 ↑	49 ↑	/	g/L	20–45
Alb/Glob	/	/	/	0.5 ↓	0.5 ↓	0.6 ↓	/	g/L	1.5–2.5

Remark: “↑” denotes values above the upper limit of the reference range; “↓” denotes values below the lower limit of the reference range. “/” means test was not performed.

**Table 2:** Mean ± SD optical density (OD) and median of antibodies in the affected horse.

	Group 1 Baseline	Group 2 Follow-Up
Number of horses	1	1
Mean OD ± SD	0.530 ± 0.073	0.675 ± 0.106 *
Median	0.569	0.704

* Statistically significant (*p* < 0.05), compared to baseline.

**Table 3:** Mean ± SD optical density (OD) and median of antibodies in different horse breeds.

	Group 3 Healthy Warmblood Horses	Group 4 Lymphedematous Warmblood Horses	Group 5 Healthy Draft Horses
Number of horses	4	4	4
Mean OD ± SD	0.332 ± 0.041	0.436 ± 0.044 *	0.515 ± 0.014 *
Median	0.325	0.420	0.516

* Statistically significant (*p* < 0.05), compared to healthy warmblood.

## Data Availability

The original contributions presented in this study are included in the article. Further inquiries can be directed at the corresponding author.

## Supplementary Materials

The following supporting information can be downloaded at: https://www.mdpi.com/article/10.3390/vetsci12060574/s1, Figure S1: Phylogenetic analysis of *P. asiatica*; Figure S2: Phylogenetic analysis of *E. Hormaechei*; Table S1: Antimicrobial susceptibility test results.
