# Supplementary material for: Pseudomonas-Enterobacter Co-Infection Drives Cellulitis and Lymphangitis in Equines: A Case Report
Source: Vet Sci. 2025 Jun 11;12(6):574. doi: 10.3390/vetsci12060574 (PMC12197406; doi:10.3390/vetsci12060574)

Antimicrobial Susceptibility Test Results

Strain ID/Sample No.: sample1 *P.asiatica*

Date Tested: 29/10/2024

Test Method: Microbiological cultivation+Mass spectrometry+Minimum Inhibitory Concentration(MIC)/Kirbv-Bauer disk diffusion(K-B)

Drug Resistance Mechanism: /

Laboratory: ZOETIS REFERENCE LABORATORIES (Shanghai, China)

Antimicrobial Agent	Test Method	Test Result	Interpretation
**Cefovecin**	MIC	≥8 μg/mL	R
**Imipenem**	MIC	0.5 μg/mL	S
**Gentamicin**	MIC	≤1 μg/mL	S
**Marbofloxacin**	MIC	≤0.5 μg/mL	S
**Tetracycline**	MIC	2 μg/mL	S
**Sulfamethoxazole/Trimethoprim (SMZ-TMP)**	MIC	≥320 μg/mL	R
**Ceftiofur**	MIC	≥8 μg/mL	R
**Amikacin**	MIC	≤2 μg/mL	S
**Enrofloxacin**	MIC	1 μg/mL	I
**Doxycycline**	MIC	1 μg/mL	S
**Chloramphenicol**	MIC	≥64 μg/mL	R

***S = Susceptible; I = Intermediate; R= Resistant**

The selection of antimicrobial agents and breakpoints in this susceptibility testing report is primarily based on the **CLSI VET01S Performance Standards for Antimicrobial Disk and Dilution Susceptibility Tests for Bacteria Isolated From Animals**, along with the **CLSI M100 Performance Standards for Antimicrobial Susceptibility Testing**.

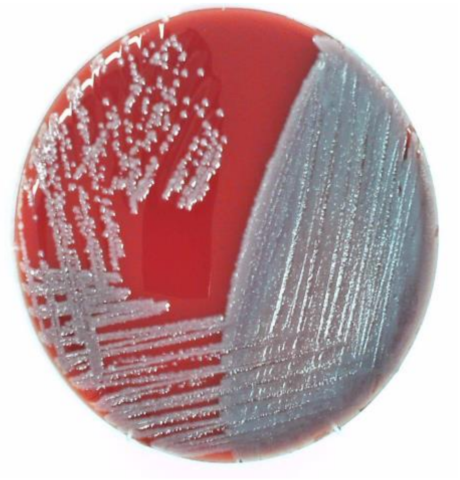

Drug Sensitive Test Results

Strain ID/Sample No.: Sample 2. *E.hormaechei*

Date Tested: 29/10/2024

Test Method: Microbiological cultivation+Mass spectrometry+Minimum Inhibitory Concentration(MIC)/Kirbv-Bauer disk diffusion(K-B)

Drug Resistance Mechanism: Bacteria producing extended-spectrum β-lactamases (ESBLs)

Laboratory: ZOETIS REFERENCE LABORATORIES (shanghai)

Antimicrobial Agent	Test Method	Test Result	Interpretation
**Amoxicillin/Clavulanate**	MIC	≥32 μg/mL	R
**Cefalotin**	MIC	≥64 μg/mL	R
**Cefovecin**	MIC	≥8 μg/mL	R
**Imipenem**	MIC	≤0.25 μg/mL	S
**Gentamicin**	MIC	≤1 μg/mL	S
**Enrofloxacin**	MIC	2 μg/mL	I
**Pradofloxacin**	MIC	2 μg/mL	R
**Tetracycline**	MIC	≥16 g/mL	R
**Sulfamethoxazole/Trimethoprim (SMZ-TMP)**	MIC	≥320 μg/mL	R
**Cefalexin**	MIC	≥64 μg/mL	R
**Cefpodoxime**	MIC	≥8 μg/mL	R
**Ceftiofur**	MIC	≥8 μg/mL	R
**Amikacin**	MIC	≤2 μg/mL	S
**Neomycin**	MIC	≤2 μg/mL	S
**Marbofloxacin**	MIC	≤0.5 μg/mL	S
**Doxycycline**	MIC	≥16 μg/mL	R
**Chloramphenicol**	MIC	≥64 μg/mL	R
**Ceftazidime**	KB	6 mm	R
**Levofloxacin**	KB	17 mm	I
**Minocycline**	KB	6 mm	R
**Ceftriaxone**	KB	6 mm	R
**Meropenem**	KB	28 mm	S
**Piperacillin/Tazobactam**	KB	23 mm	I

***S = Susceptible; I = Intermediate; R= Resistant**

The selection of antimicrobial agents and breakpoints in this susceptibility testing report is primarily based on the **CLSI VET01S Performance Standards for Antimicrobial Disk and Dilution Susceptibility Tests for Bacteria Isolated From Animals**, along with the **CLSI M100 Performance Standards for Antimicrobial Susceptibility Testing**.

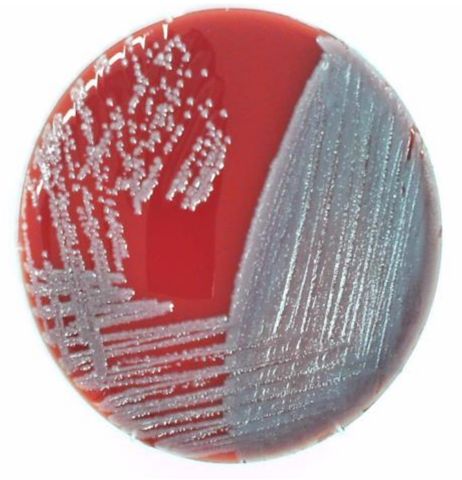

Supplement: Supplementary file 1 [file vetsci-12-00574-s001.zip › Table S1. antimicrobial susceptibility test results.pdf]
