# Supplementary figures and images for: Pseudomonas-Enterobacter Co-Infection Drives Cellulitis and Lymphangitis in Equines: A Case Report
Source: Vet Sci. 2025 Jun 11;12(6):574. doi: 10.3390/vetsci12060574 (PMC12197406; doi:10.3390/vetsci12060574)

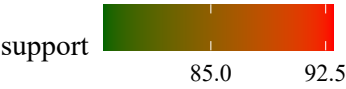

Bootstrap Percentage(BP) ● BP < 70 ● 70 ≤BP < 90 ● 90 ≤BP ≤ 100

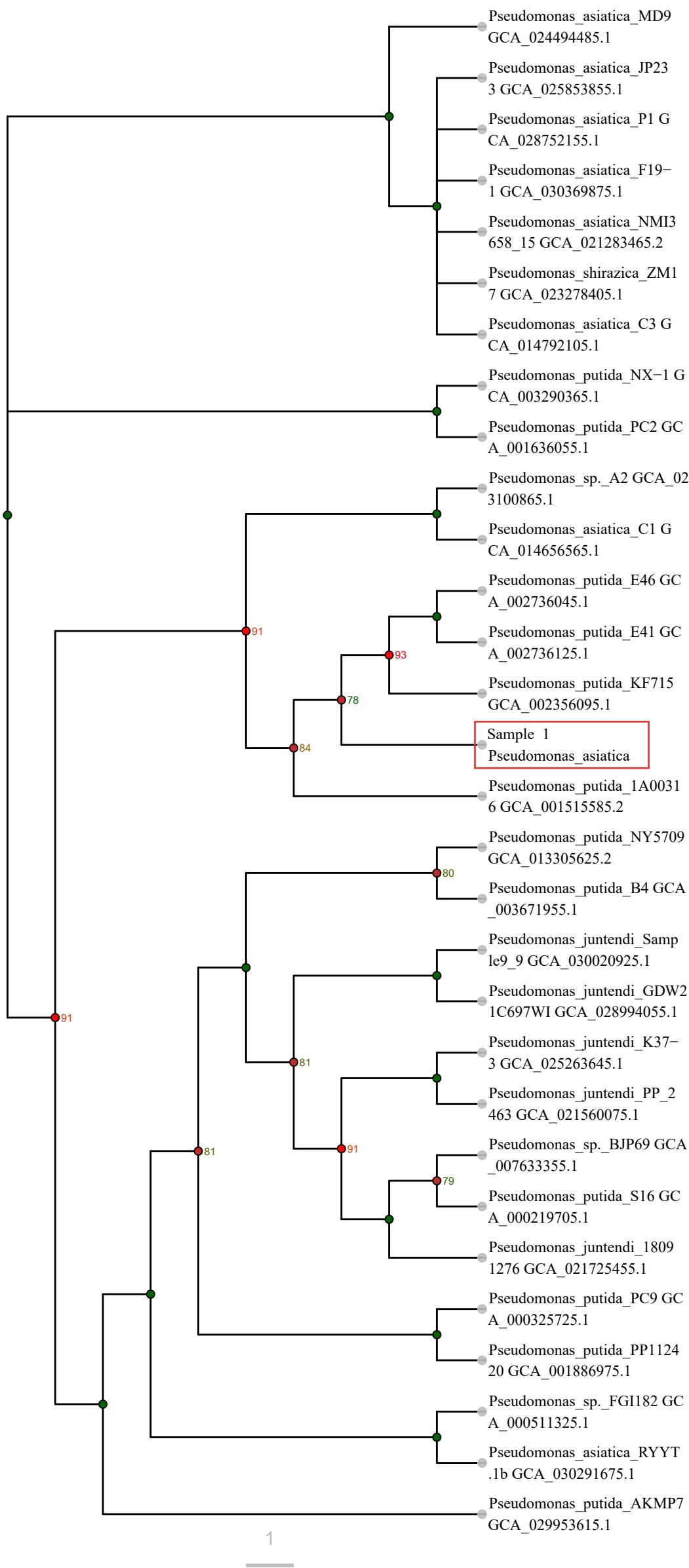

Supplement: Supplementary file 1 [file vetsci-12-00574-s001.zip › Figure S1. P.asiatica-sample 1.pdf]

Bootstrap Percentage(BP)    ● BP < 70    ● 70 ≤BP < 90    ● 90 ≤BP ≤ 100

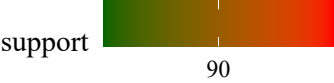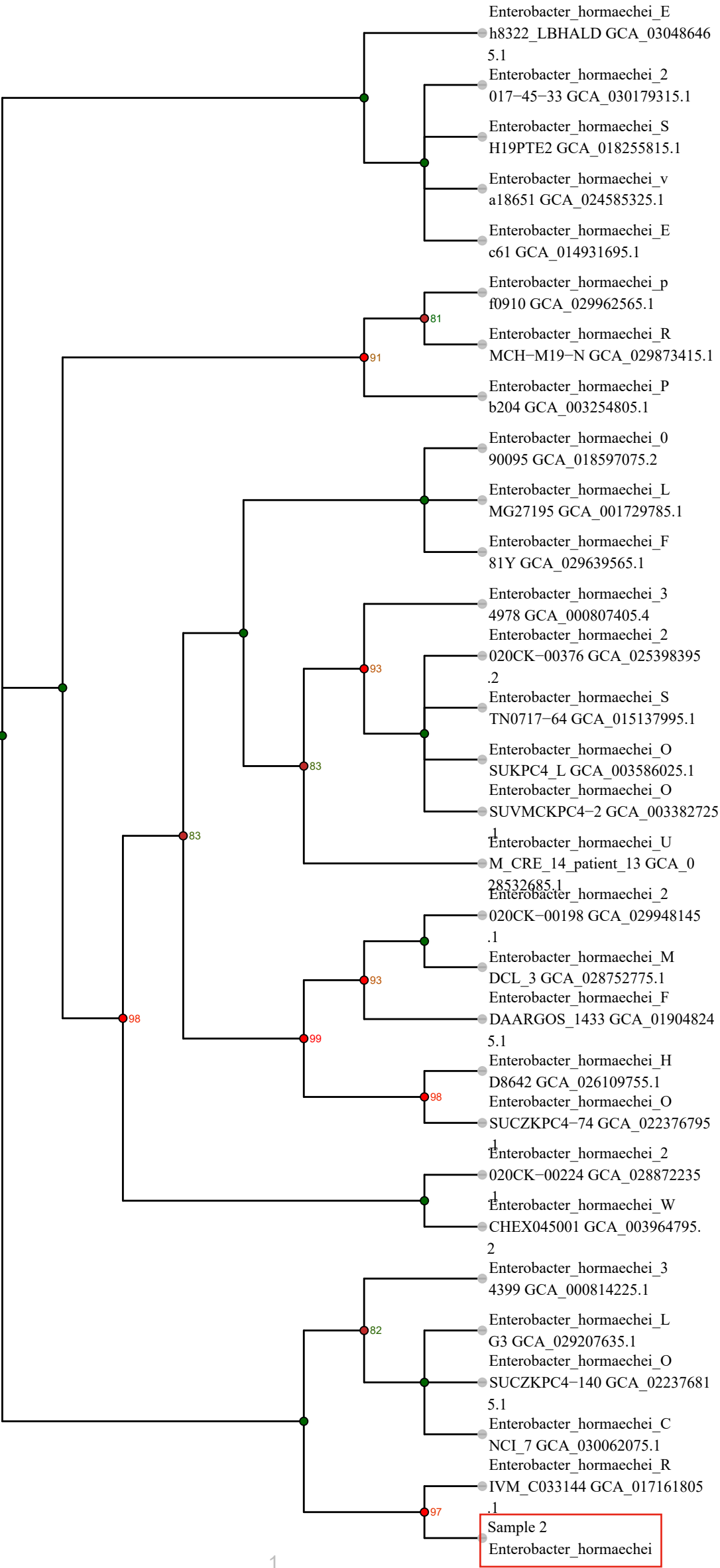

Supplement: Supplementary file 1 [file vetsci-12-00574-s001.zip › Figure S2. E.hormaechei-sample2.pdf]
